# Sinomenine Attenuated Capsaicin-Induced Increase in Cough Sensitivity in Guinea Pigs by Inhibiting SOX5/TRPV1 Axis and Inflammatory Response

**DOI:** 10.3389/fphys.2021.629276

**Published:** 2021-08-05

**Authors:** Jian-Ling Ma, Kun Ji, Li-Qing Shi, Niu-Niu Li, Li-Yun Wang, Shang-Juan Dong, Yan-Xia Zhang, Shao-Hui Wen, Xue-Mei Liu, Ying Wang, Jing-Yue Luo

**Affiliations:** ^1^Department of Respiratory, Dongfang Hospital, Beijing University of Chinese Medicine, Beijing, China; ^2^Laboratory Center, Dongfang Hospital, Beijing University of Chinese Medicine, Beijing, China; ^3^Beijing University of Chinese Medicine, Beijing, China

**Keywords:** cough sensitivity, SOX5/TRPV1, SP, NKA, guinea pigs

## Abstract

**Background:**

Chronic cough is a common complaint which affects a large number of patients worldwide. Increased cough sensitivity is a very important cause of chronic persistent cough. However, there are limited clinical diagnosis and treatment for increased cough sensitivity. Transient receptor potential vanilloid-1 (TRPVl) is a member of the transient receptor potential (TRP) family of channels which is very closely associated with respiratory diseases. However, the mechanism through which TRPV1 that influences downstream events is still poorly understood.

**Results:**

Capsaicin induced increase in cough sensitivity by upregulating the protein level of TRPV1, leading to the secretions of Substance P and neurokinin A which stimulated neurogenic inflammation. However, sinomenine, a component of traditional Chinese medicine, significantly attenuated the capsaicin-induced cough by inhibiting the expression of TRPV1 in guinea pigs. In addition, capsaicin increased the expression of SOX5 which mediated the transcriptional upregulation of TRPV1. However, pretreatment with sinomenine reduced the expression of SOX5.

**Conclusion:**

These results indicate that capsaicin induced increase in cough sensitivity by activating neurogenic inflammation, while sinomenine attenuated the increase in cough sensitivity by inhibiting the expressions of SOX5 and TRPV1 in guinea pigs. This finding may provide a novel target for the treatment of aggravated cough sensitivity.

## Background

Sinomenine is a traditional Chinese medicine purified from the roots of the climbing plant *Sinomenium acutum*. Sinomenine was originally found to be effective in treating rheumatism in Japan since the early 1930s (Yamasaki, 1976). It can also function as an immune suppressor since it inhibits lymphocyte proliferation and suppresses the synthesis of B-cell antibodies (immunoglobulin G) in cells and animals (He et al., 2005; Feng et al., 2007). In addition to its anti-rheumatic properties, recent studies also demonstrated the efficacy of sinomenine in alleviating pain (Jiang et al., 2020). Besides, several studies have found that sinomenine plays a vital role in varied biological processes such as cell proliferation, apoptosis, metastasis, and angiogenesis (Ou et al., 2011; Feng et al., 2019; Zhang et al., 2019). It has been reported that sinomenine attenuated airway inflammation by inhibiting the expressions of TGF-β1 and CTGF (Bao et al., 2016). However, the mechanism through which sinomenine prevents airway inflammation is still poorly understood.

Cough is caused by inflammation or chemical stimulation of the trachea or pleura. Although cough exerts a protective effect by clearing away foreign bodies from the respiratory tract, it always brings pain to the patients (Morice and Shanks, 2017). Cough is a common complaint in clinics, especially chronic cough without obvious abnormality in chest imaging examination. There are several types of chronic cough: cough variant asthma, upper airway cough syndrome, gastroesophageal reflux cough, eosinophilic bronchitis, refractory cough, and cough hypersensitivity syndrome, and they account for about 70–95% of chronic cough (Chung and Pavord, 2008; Mazzone et al., 2018). Since the etiology of chronic cough is difficult to determine, there are high rates of clinical misdiagnosis and mistreatment, leading to serious problems for patients (Guilleminault et al., 2019). Aggravated cough sensitivity is an important factor for the development of chronic cough.

Airway neurogenic inflammation is an important mechanism involved in accentuated cough sensitivity, and its persistence is a very important cause of chronic persistent cough (Morice and Shanks, 2017; Chen et al., 2018; Zhang et al., 2018). Neuropeptide is a major mediator of airway neurogenic inflammation. Neuropeptides participate in neurogenic inflammation in many ways, such as stimulation of cough receptors, promotion of mucus secretion, enhancement of vascular permeability, promotion of cholinergic neurotransmitter delivery, contraction of tracheal smooth muscle, and activation of inflammatory cells. Substance P (SP) and neurokinin A (NKA) are the most important factors that regulate neurogenic inflammation (Chen et al., 2017). They are produced by ganglion cells and released in the periphery through the sensory nerve endings of the airway. They act by promoting mast cell degranulation, releasing a large number of inflammatory mediators such as histamine, kinins, prostaglandins and leukotrienes, and promoting monocytes to secrete inflammatory factors such as IL-1, IL-6, and TNF-a, thereby further aggravating tissue inflammatory response (Cuesta et al., 2002; Yamaguchi et al., 2017).

One of the most thoroughly studied members of the transient receptor potential cation channel protein (TRP) family is transient receptor potential vanilloid-1 (TRPV1) (Bevan et al., 2014). It is distributed mainly in mammalian sensory nerve fibers such as the vagus and sensory nerve fibers of dorsal root ganglion, especially unmyelinated C-fibers. It is believed that TRPV1 plays an important role in cough reflex (Lv et al., 2016). It controls the release of SP and NKA, and induces neurogenic inflammation, leading to increased airway sensitivity (Geppetti et al., 2006; Marrone et al., 2017). These studies show that TRPV1 channel promotes inflammation, especially neurogenic inflammation, through a mechanism involving complex interaction with SP and other neuropeptides.

In this study, it was found that increased cough sensitivity was stimulated *via* neurogenic inflammation in capsaicin-treated guinea pigs. Sinomenine inhibited the expression of TRPV1 and secretions of SP and NKA, and attenuated increased cough sensitivity. In addition, capsaicin administration increased the protein level of TRPV1, which was transcriptionally regulated by SOX5. Sinomenine pretreatment significantly inhibited the expressions of SOX5 and TRPV1, and suppressed the secretions of SP and NKA in guinea pigs.

## Materials and Methods

### Experimental Animals

The experimental protocols were conducted in accordance with the National Institutes of Health guidelines. This study was reviewed and approved by the Institutional Animal Care and Use Committee of Beijing University of Chinese Medicine. Male guinea pigs (200–250 g) were provided by Cyagen Biosciences, Suzhou, China. They were randomly divided into the following groups: control, capsaicin (Solarbio, Beijing), capsaicin + sinomenine, capsaicin + si-SOX5, and capsaicin + sinomenine + SOX5. All guinea pigs were kept in IVC rearing cage in SPF animal rooms with 12 h light/12 h dark cycle at temperature of 20–26°C. There were two guinea pigs per cage, and each cage had autoclaved bedding materials (Cyagen Biosciences, Suzhou, China).

**FIGURE 1:**
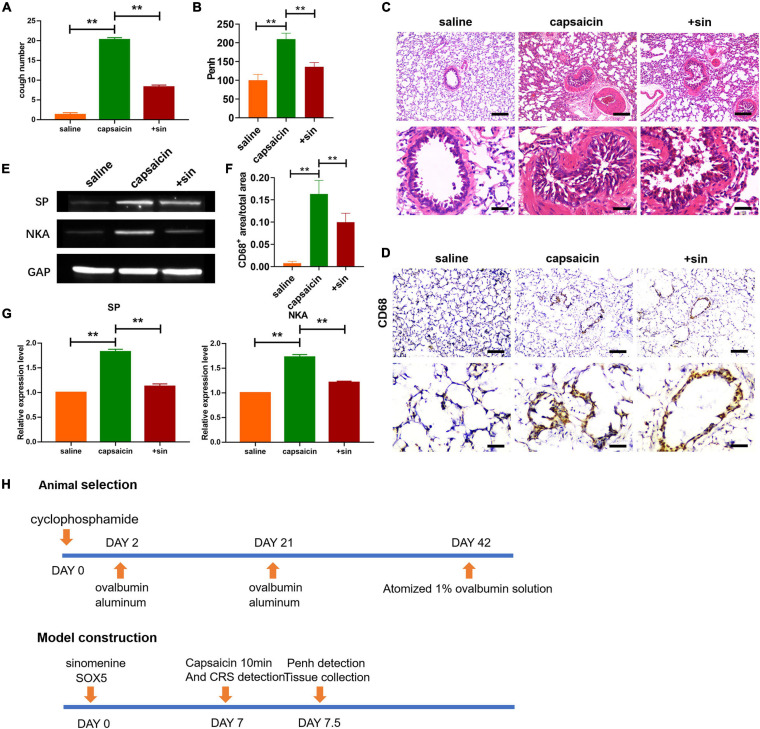
Sinomenine treatment attenuated capsaicin-induced cough sensitivity in guinea pigs. **(A)** Cough reflex sensitivity (CRS) in guinea pigs. Sinomenine inhibited the cough induced by the capsaicin. Data are mean ± SEM; one-way ANOVA was used for the statistical analysis. **(B)** Sinomenine reduced the capsaicin-induced increase in Penh. Data are mean ± SEM; one-way ANOVA was used for the statistical analysis. **(C)** H&E staining of lung tissues showing the levels of infiltration by inflammatory cells. Scale bars represent 50 and 100 μm. **(D,F)** CD68 staining of lung tissues from guinea pigs treated with saline, capsaicin, or sinomenine. Scale bars represent 50 and 100 μm. **(E,G)** Western blot assay of the expression levels of SP and NKA in trachea and lung tissues of guinea pigs. Data are mean ± SEM; one-way ANOVA was used for the statistical analysis. **(H)** Scheme for the screening of guinea pigs and construction of cough model. **P < 0.01; *n* = 7.

The guinea pig with enhanced cough sensitivity model was established as described earlier (Ji et al., 2017). In order to sensitize guinea pigs, cyclophosphamide (Solarbio, Beijing) was injected into the abdominal cavity at a dose of 30 mg/kg. Two days later, 2 mg ovalbumin and 100 mg aluminum hydroxide (Solarbio, Beijing) were injected into the abdominal cavity. After 3 weeks, 0.01 mg ovalbumin (Solarbio, Beijing) and 100 mg aluminum hydroxide were injected into the abdominal cavity. Three weeks after the enhancement of immunization, the guinea pigs were placed in self-made aerosol box, and stimulated with 1% ovalbumin solution atomized with Devilliis 646 atomizer for 90 s at an outflow rate of 0.037 ml/min). The number of coughs within 3 min was counted. Guinea pigs with cough reflex due to stimulation were considered as successful model of increased cough sensitivity (Figure 1H).

In all experiments, capsaicin was used to induced cough in guinea pigs. The guinea pigs were placed in transparent plastic bottles and each animal was exposed to capsaicin (50 μmol/L, 1 mL) vapor *via* an ultrasonic atomizer for 10 min. In the sinomenine-treated group, the guinea pigs were pretreated with sinomenine at a dose of 0.5/100 g body weight/day through intragastric administration for 7 days. The crude drug dose of sinomenine was 0.5 g sinomenine herbal powder/100 g body weight of guinea pig/day. Recombinant human SOX5 (2 mg/kg, 0.5 mg/ml in PBS) was purchased from R&D, and was administered *via* atomization inhalation with capsaicin (Figure 1H). Adenovirus overexpressing TRPV1 was administered *via* atomization inhalation 2 days before capsaicin treatment.

All animals were sacrificed under intraperitoneal anesthesia with 3% pentobarbital sodium at a dose of 90 mL/kg bwt, and the trachea and lung tissues were excised for use in follow-up studies. Portions of the lung tissues were fixed in 4% paraformaldehyde (Solarbio, Beijing) for 3 days, and embedded in paraffin for H&E and IHC staining. The other portions of lung tissues were used for the extractions of total RNA and protein. The sample size (*n*) for each experimental group/condition was 7 (*n* = 7).

### Cough Reflex Sensitivity Analysis

Single chamber unrestricted body scanner (Buxco, United States) was used to determine cough reflex sensitivity (CRS) level of guinea pigs. The total number of cough was counted in 10-min duration, and it was used to reflect CRS level. Changes in air flow signal were measured using Buxco system. The area under the curve (V2) and the half peak conversion time of the air flow were calculated to judge whether it was cough, sneeze, or other irregular activities. Atomized capsaicin (50 μmol/L, 1 mL) was used to induce cough for 10 min, and cough count was recorded.

### Airway Responsiveness Analysis

After determination of CRS (12 h), non-invasive animal lung function detector FinePointe^TM^ NAM (Buxco, United States) was used to determine enhanced pause (Penh) level. Aerosolized methacholine (200 mg/L, 100 μL) was used as a stimulant to induce airway response. Average value of Penh was recorded and converted to the percentage Penh value for saline group, expressed as Penh %, as an evaluation index of airway responsiveness.

### Cell Culture and Transfection

Primary mouse hippocampal neuron cells (HNC) were isolated as described below: Neonatal guinea pigs were anesthetized with isoflurane and sterilized using 75% ethanol. Brain tissue was cut into small sections (1mm3) and digested with pancreatin (Beyotime Biotechnology, Beijing). The digested cell suspensions were centrifuged at 1000 rpm, and the cell pellet was resuspended in DMEM containing 10% FBS. The neuronal cells were cultured in cell incubator under 5% CO_2_ for 48 h.

Next, Si-SOX5 and TRPV1 overexpression plasmids were produced by RiboBio (Guangzhou, China). Neuronal cells were transfected according to the manufacturer’s protocol using X-treme GENE siRNA transfection reagent (Roche, Germany). Six hours later, the transfection medium was changed to culture medium containing 10% FBS, and the cells were cultured for 48 h. In the capsaicin-treated groups, 50 μM capsaicin was administered for 3 h. For SOX5-related groups, cells were treated with SOX5 (2 ng/ml) for 24 h before capsaicin treatment.

### Real Time-PCR

Total RNA was extracted from cells and tissue samples with TRIzol reagent (Invitrogen) according to the manufacturer’s protocol. The samples were treated with DNase I before reverse transcription into cDNA in line with the manufacturer’s protocol (iScript, Bio-Rad). Quantitative Real-Time PCR was performed using LightCycler 480 (Roche) and SYBR Green Master Mix (Roche) with corresponding primers in line with standard protocol. All assays were done at least in duplicate, and in a total of at least two independent assays. The relative expression levels were calculated using the 2^–ΔΔCt^ method. Data were analyzed by GraphPad 7. The sequences of primers used were: SOX5: Forward: CTGCCGCCATTGATGATTCC; Reverse: CCAGCCTTGTAGCTGAAACCA; TRPV1: Forward: CCGGCTTTTTGGGAAGGGT; Reverse: GAGACAGGTAG GTCCATCCAC.

### Western Blot Assay

The *in vivo* protein expression levels of SP and NKA in trachea and lung tissues were determined using standard immunoblotting protocol^1^. The membranes were scanned with Odyssey Infrared Scanning System (Gene Co., Ltd., Hong Kong, China), and the blot results were analyzed using ImageJ software. The primary antibodies used were CD68 and GAPDH antibodies (Proteintech Group, Wuhan, China), as well as SOX5 and TRPV1, SP and NKA primary antibodies (Cell Signaling Technology, Danvers, MA, United States). The secondary antibodies (IRDye700/800 mouse and rabbit) were products of LICOR (Lincoln, NE, United States).

### Luciferase Reporter Assay

The psiCHECK-2 luciferase reporter plasmid (Transgen, Beijing) was inserted into the wild-type TRPV1 promotor or mutant TRPV1 promotor sequences that contained the putative binding sites of SOX5 to construct SOX5-WT or SOX5-Mut expression plasmid which was transfected with reporter vectors into neuronal cells. The cells were collected 48 h post-transfection and lysed, and luciferase activity (Promega) was assayed in the lysates.

### Immunofluorescence Staining

Neuronal cells were plated in a 24-well cell culture plate. After capsaicin treatment, the cells were washed with PBS and fixed with 4% paraformaldehyde, followed by permeabilization with 0.2% Triton-X-100 solution in PBS. Next, the cells were blocked using goat serum, and incubated with SOX5 antibody at 4°C overnight, followed by incubation with FITC-conjugated goat anti-mouse antibodies for 1 h. After three washes with PBS, the cells were incubated with DAPI. Frozen sections of guinea pig were fixed in 4% paraformaldehyde, washed using PBS, and permeabilized with 0.5% Triton X-100. After washing three times, the blocked sections were treated with 50% goat serum. Then, the sections were incubated with SOX5 antibody overnight, incubated with secondary antibody, and stained with DAPI. Immunofluorescence was measured using an IX73 fluorescence microscope (Olympus, Valley, PA, United States).

### Immunohistochemical Staining

Paraffin sections of lung tissue were dewaxed in xylene and descending series of ethanol. The sections were permeabilized using 0.5% Triton X-100. After washing three times, they were blocked with 50% goat serum. Then, the sections were incubated with SOX5 primary antibody overnight, followed by incubation with secondary antibody for 1 h at room temperature. Thereafter, the sections were DAPI-stained for 5 min at room temperature. The sections were photographed with light scope under an IX73 fluorescence microscope (Olympus, Valley, PA, United States), and analyzed using ImageJ software.

### Measurement of Intracellular Calcium Levels

Hanks Balanced Salt Solution (HBSS) was used to dilute BALB cellProbe F3 solution 2000 times. This was used as working solution. The cells were added to 1 mL of working solution instead of culture media, and cultured at 37°C for 20 min. Then, the same volume of HBSS media containing 1% FBS was added to cells and incubated at 37°C for 40 min. Thereafter, the cells were digested, centrifugated and resuspended in 1 mL of HBSS, followed by measurement of fluorescence intensity (Promega).

### Statistical Analysis

All data are presented as a mean ± SEM. Statistical analyses were performed using the GraphPad Prism 8 software. Two-group comparison was done with two-tailed Student’s *t*-test, while multi-group comparison was done with one-way ANOVA. Values of *p* < 0.05 were considered as indicative of statistical significance.

## Results

### Sinomenine Attenuated Capsaicin-Induced Increase in Cough Sensitivity by Inhibiting Neurogenic Inflammation

To determine the effect of sinomenine on increased cough sensitivity induced by neurogenic inflammation, a guinea pig model of increased cough sensitivity was used. As shown in Figure 1A, capsaicin treatment increased the CRS of guinea pigs. In contrast, pretreatment with sinomenine suppressed CRS. Furthermore, compared to saline group, capsaicin treatment increased the Penh level (Figure 1B). However, sinomenine significantly reduced the level of Penh (Figure 1B). These data suggest that sinomenine can significantly attenuate capsaicin-induced high sensitivity of cough in guinea pigs. Results from H&E staining showed that sinomenine pre-administration reversed capsaicin-induced increase in inflammatory cell infiltration (Figure 1C). Immunohistochemical staining showed that atomized capsaicin treatment led to increased infiltration by monocytes/macrophages. However, sinomenine significantly reduced the level of CD68-positive monocytes/macrophages (Figures 1D,F). SP and NKA are two kinds of sensory neuropeptides which act as important inflammatory mediators that stimulate mast cells, affect the production of inflammatory factors by T-lymphocytes and B-lymphocytes, induce neurogenic inflammation, increase airway sensitivity, and cause persistent chronic cough (Wiesenfeld-Hallin and Xu, 1993; Bhatia, 2015; Malhotra, 2016; Mashaghi et al., 2016). Western blot analysis was performed to measure the expression levels of SP and NKA. As shown in Figures 1E,G, capsaicin upregulated the expressions of SP and NKA, while sinomenine pretreatment suppressed the expressions of SP and NKA. These data indicate that sinomenine inhibited capsaicin-induced increase in cough sensitivity in guinea pigs through mitigation of neurogenic inflammation.

### Sinomenine Inhibited the Neurogenic Inflammation by Suppressing the Expression of TRPV1 in Neuronal Cells

**FIGURE 2:**
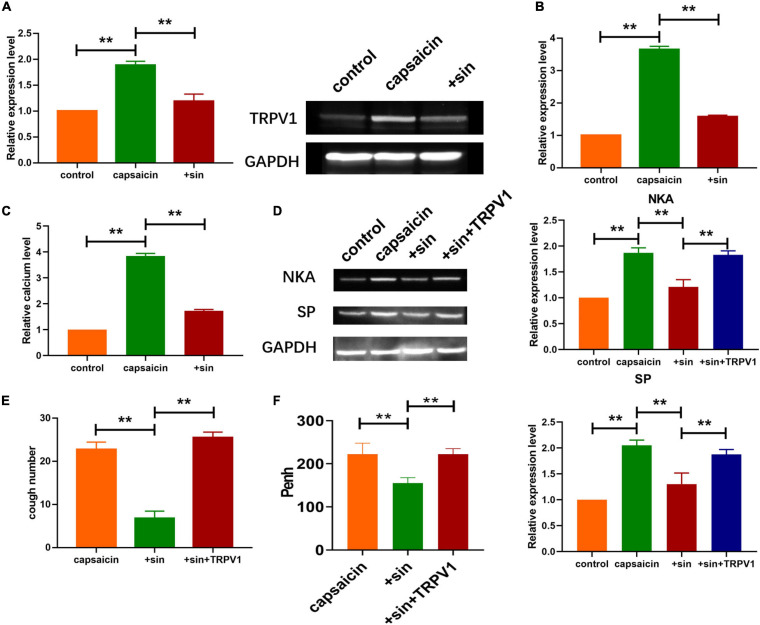
Sinomenine inhibited the expression of TRPV1 induced by capsaicin in neuronal cell. **(A)** Expression level of TRPV1 in neuronal cells, as measured using western blot analysis. Data are mean ± SEM; one-way ANOVA was used for statistical analysis. **(B)** qRT-PCR analysis showing the mRNA level of TRPV1 in neuronal cells. Data are mean ± SEM; one-way ANOVA was used for statistical analysis. **(C)** Intracellular calcium levels. Sinomenine inhibited capsaicin-induced calcium uptake in neuronal cells. Data are mean ± SEM; one-way ANOVA was used for statistical analysis. **(D)** Protein expression levels of SP and NKA were reduced by sinomenine and elevated after TRPV1 overexpression in neuronal cells. **(E)** CRS levels showing that effect of sinomenine was inhibited after TRPV1 administration. Data are mean ± SEM; one-way ANOVA was used for statistical analysis. **(F)** Effect of TRPV1 administration on Penh. Data are mean ± SEM; one-way ANOVA was used for statistical analysis. **P < 0.01; *n* = 7.

Growing evidence indicate that TRPV1 plays important role in cough and other airway diseases (Lee et al., 2011; Millqvist, 2016). To determine if TRPV1 was involved in the regulation of cough by sinomenine, western blot was performed to determine the expression levels of TRPV1 in trachea and lung tissues. Compared with saline group, atomized capsaicin treatment significantly elevated the protein expression of TRPV1. In contrast, sinomenine pretreatment reduced the protein level of TRPV1 (Figure 2A). In addition, mRNA expression level of TRPV1 is shown in Figure 2B. Previous studies indicated that TRPV1 regulated intracellular calcium levels. As shown in Figure 2C, a specific calcium probe was used to determine changes in intracellular calcium levels after atomized capsaicin treatment as described earlier (Barreto-Chang and Dolmetsch, 2009). Results showed that sinomenine pretreatment significantly reduced the capsaicin-induced upregulation of calcium level. Furthermore, western blot analysis showed that overexpression of TRPV1 abolished the inhibitory effect of sinomenine on expressions of SP and NKA (Figure 2D). Moreover, overexpression of TRPV1 reversed the protective effect of sinomenine pretreatment and increased the levels of Penh while reducing CRS level in the guinea pigs (Figures 2E,F).

### SOX5 Mediated Capsaicin-Induced Upregulation of TRPV1 as a Transcription Factor

**FIGURE 3:**
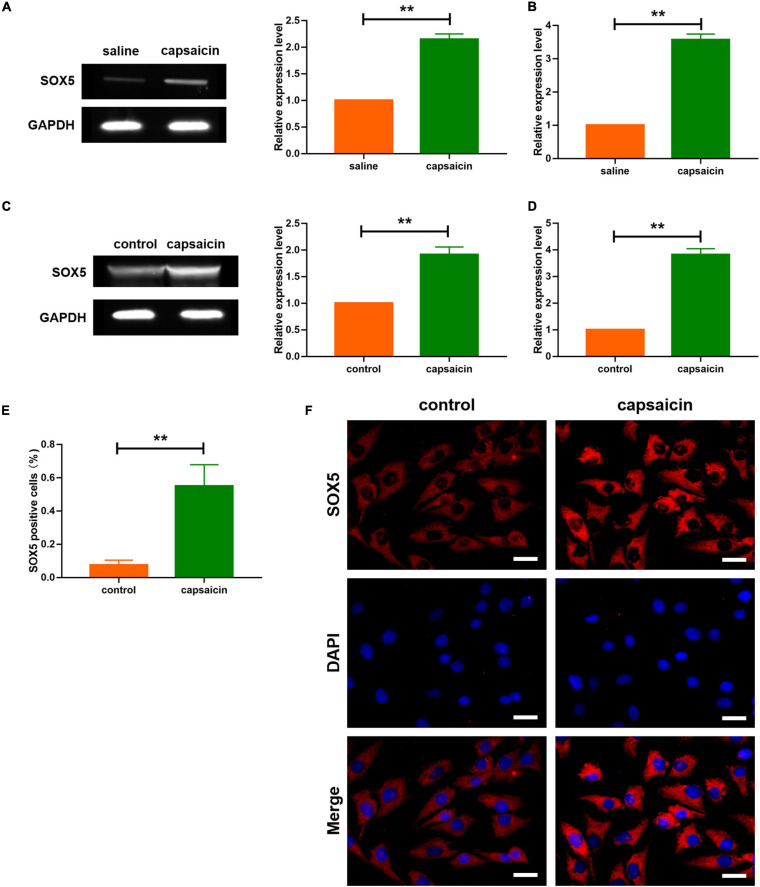
Effect of capsaicin on Sox5 in guinea pig and neuronal cells. **(A)** Expression level of SOX5 after capsaicin treatment in guinea pigs. Data are mean ± SEM; two-tailed *t*-test was used for statistical analysis. **(B)** mRNA expression level of SOX5 in guinea pigs after capsaicin treatment. Data are mean ± SEM; two-tailed *t*-test was used for statistical analysis. **(C)** Expression level of SOX5 after capsaicin treatment in neuronal cells. Data are mean ± SEM; two-tailed *t*-test was used for statistical analysis. **(D)** mRNA expression level of SOX5 in neuronal cells after capsaicin treatment. Data are mean ± SEM; two-tailed *t*-test was used for statistical analysis. **(E)** SOX5 level, as determined using immunofluorescence, and SOX5-positive staining in trachea and lung tissues of guinea pigs. Data are mean ± SEM; two-tailed *t*-test was used for statistical analysis. **(F)** Immunostaining analysis showing that capsaicin upregulated the expression of SOX5 in neuronal cells. Scale bars represent 100 μm. **P < 0.01; *n* = 7.

**FIGURE 4:**
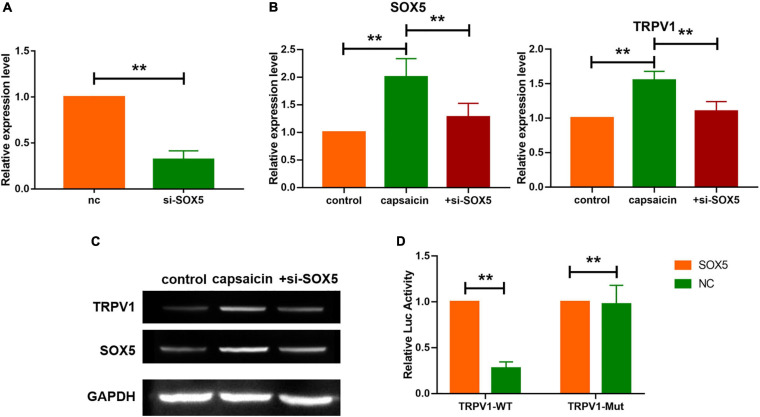
Sox5 mediated the expression of TRPV1 in neuronal cells in response to capsaicin administration. **(A)** mRNA level of SOX5 in neuronal cells. Data are mean ± SEM; two-tailed *t*-test was used for statistical analysis. **(B,C)** Upregulation of TRPV1 was inhibited after si-SOX5 transfection. Data are mean ± SEM; one-way ANOVA was used for statistical analysis. **(D)** SOX5 induced the expression of TRPV1 gene, as determined using luciferase assay. Data are mean ± SEM; one-way ANOVA was used for statistical analysis. **P < 0.01; *n* = 7.

It is known that SOX5, a transcription factor expressed widely during development in several tissues, is a member of Sry-related HMG-box family (Zhang and Liu, 2017; Hu et al., 2018). Prediction data from Genecards^2^ showed a binding site of SOX5 on the sequence of TRPV1. Firstly, the expression levels of SOX5 in neuronal cells and trachea and lung tissues were determined. Western blot analysis showed that the protein level of SOX5 was increased in trachea and lung tissues, and in neuronal cells after capsaicin treatment (Figures 3A,C). In addition, capsaicin administration upregulated the transcription levels of SOX5 gene in trachea and lung tissues, and in neuronal cells (Figures 3B,D). Furthermore, immunological staining showed that capsaicin treatment significantly elevated the expression level of SOX5 (Figures 3E,F). Then, the efficiency of SOX5 knockdown was determined. The si-RNA of SOX5 significantly decreased the mRNA level of SOX5 (Figure 4A). Capsaicin was added to neuronal cells which were transfected with si-SOX5 to block the expression of SOX5. Western blot analysis showed that capsaicin increased the protein level of TRPV1 (Figures 4B,C). However, siRNA interference of SOX5 significantly reduced the expression of TRPV1, suggesting that SOX5 is an important regulator of TRPV1. Moreover, luciferase assay indicated that SOX5 had no effect on the vector carrying mutant binding site of TRPV1 (TRPV1-Mut) which inhibited the activity of wild-type TRPV1 luciferase vector (TRPV1-WT) (Figure 4D). These results indicate that SOX5 transcription regulated TRPV1 expression.

### Sinomenine Reduced Neurogenic Inflammation and Attenuated Increased Cough Sensitivity by Regulating SOX5/TRPV1

**FIGURE 5:**
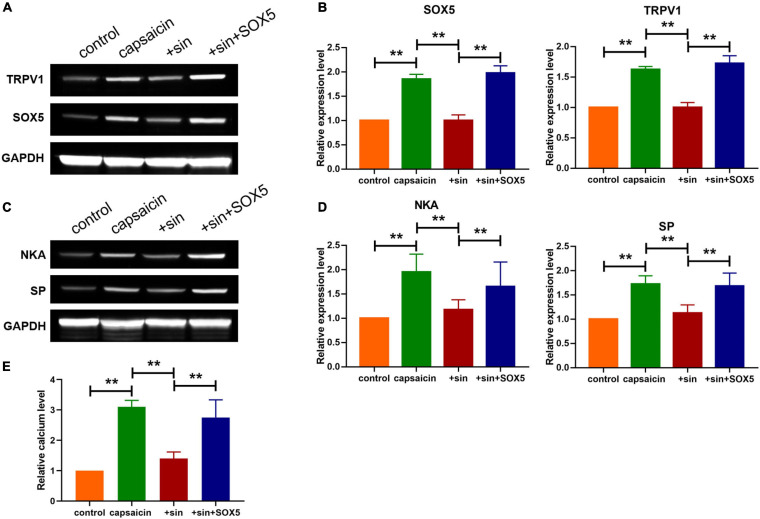
Sinomenine reduced the protein level of TRPV1 *via* downregulating SOX5 expression in neuronal cells. **(A,B)** Effect of sinomenine treatment and SOX5 administration on the protein expression levels of TRPV1 and SOX5 in neuronal cells. Data are mean ± SEM; one-way ANOVA was used for statistical analysis. **(C,D)** Effect of sinomenine treatment and SOX5 administration on the protein levels of SP and NKA in neuronal cells. Data are mean ± SEM; one-way ANOVA was used for statistical analysis. **(E)** Intracellular calcium levels in neuronal cells. Data are mean ± SEM; one-way ANOVA was used for statistical analysis. **P < 0.01; *n* = 7.

**FIGURE 6:**
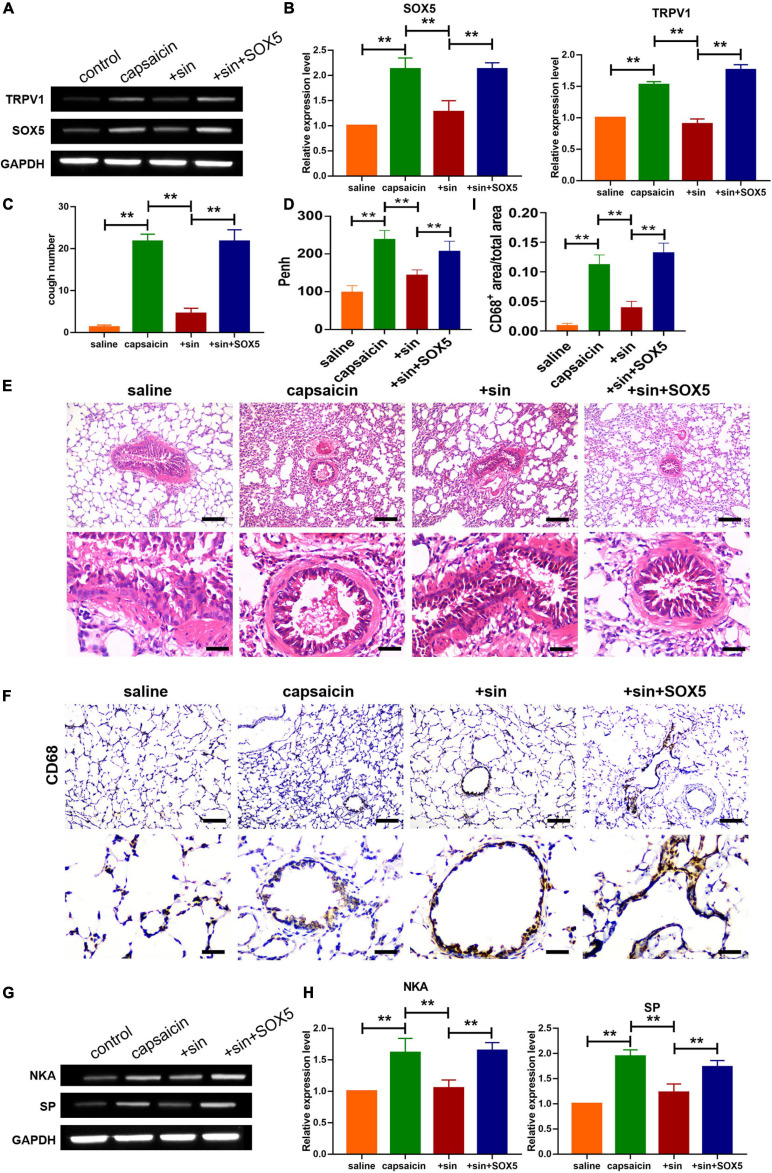
Effect of sinomenine on capsaicin-induced increase in cough sensitivity in guinea pigs, and the implication of SOX5 and TRVP1 in the process. **(A,B)** Protein expression levels of TRPV1 and SOX5 in guinea pigs, as assayed using western blotting. Data are mean ± SEM; one-way ANOVA was used for statistical analysis. **(C)** CRS in guinea pigs showing that treatment by sinomenine and SOX5 increased the number of cough. Data are mean ± SEM; one-way ANOVA was used for statistical analysis. **(D)** SOX5 administration enhanced Penh. Data are mean ± SEM; one-way ANOVA was used for statistical analysis. **(E)** H&E staining showing levels of inflammatory cell infiltration. Scale bars represent 50 and 100 μm. **(F,I)** CD68 staining indicating that SOX5 increased the infiltration of monocytes/macrophages. Scale bars represent 50 and 100 μm. **(G,H)** Western blot analysis showing the expression levels of SP and NKA in guinea pigs. Data are mean ± SEM; one-way ANOVA was used for statistical analysis. **P < 0.01; *n* = 7.

Western blot analysis showed that sinomenine suppressed the level of SOX5, but SOX5 transfection significantly reversed the inhibitory effect of sinomenine on TRPV1 expression in neuronal cells (Figures 5A,B). Moreover, western blot analysis showed that overexpression of SOX5 abolished the effect of sinomenine and enhanced expressions of SP and NKA (Figures 5C,D). Intracellular calcium, which was reduced by sinomenine administration, was also upregulated after the forced expression of SOX5 (Figure 5E). These data indicate that sinomenine inhibited the expression of TRPV1 *via* downregulation of the protein level of SOX5 which acted as a transcription factor of TRPV1. Then, it was determined whether sinomenine regulated increased cough sensitivity in guinea pigs through SOX5/TRPV1. Western blot analysis showed that capsaicin-mediated upregulations of SOX5 and TRPV1 were attenuated after treatment of sinomenine, but these were reversed by SOX5 overexpression in lung tissues (Figures 6A,B). As shown in Figure 6C, CRS level was upregulated by capsaicin, but it was inhibited in sinomenine pretreatment group (Figure 6C). However, SOX5 attenuated the effect of sinomenine and increased CRS level (Figure 6C). Penh level was also measured. It was revealed that SOX5 significantly increased the level of Penh, but this effect was decreased by sinomenine pretreatment (Figure 6D). Besides, results from H&E staining showed that SOX5 abolished the sinomenine-induced inhibition of inflammatory cell infiltration (Figure 6E). Immunohistochemical staining analysis indicated that the number of CD68-positive monocytes/macrophages was also increased by SOX5 which reversed the inhibitory effect of sinomenine (Figures 6F,I). As shown in Figures 6G,H, the expressions of SP and NKA were also restored through SOX5 over-expression.

## Discussion

Cough is one of the most common symptoms of respiratory problems worldwide (Mathur et al., 2019). It is used as a kind of protective reflex in the body. Its function is to clear secretions and exudates from the respiratory tract, as well as substances penetrating the respiratory tract, and to clear irritant factors from the respiratory tract. It is a defensive reflex of the body aimed at preventing infection. Chronic cough is defined as a cough that lasts for more than 8 weeks. It can be caused by a variety of conditions.

Neurophysiological studies on chronic cough have shown that there is a complex reflex arc in involuntary cough. It starts from the sensory nerve of airway epithelium which is called cough receptor. Following stimulation, the nerve impulse travels along the vagus nerve, through its cell bodies in the ganglia nodosus and jugular vein ganglia to the cough center of brainstem, and causes cough reflex through the efferent nerve. Many diseases of the respiratory system induce cough. Airway inflammation is a common feature and an important pathological basis for cough. Airway inflammation may be classified into infective, allergic, and neurogenic types. Different types of inflammation may occur simultaneously and affect each other.

In studies on neurogenic inflammation, Barnes (1986) first proposed the axonal reflex mechanism theory. When the airway epithelium is damaged, the sensory nerve endings are exposed to some inflammatory mediators. The sensory neurons are stimulated to release a variety of neuropeptides through axonal reflex or dorsal root reflex and target receptors on the effector cells. These result in increased microvascular permeability and exudation of intracellular fluid, contraction of bronchi and activation of inflammatory cells (McDowell, 2000). Ultimately, there is enhanced release of inflammatory mediators and development of airway inflammatory response (Barnes, 1996). In this study, capsaicin induced infiltration of neutrophils/macrophages, indicating induction of airway inflammation. In addition, the mRNA and protein expression levels of two important mediators of airway neurogenic inflammation (SP and NKA) were increased after atomized capsaicin treatment, suggesting that the neurogenic inflammation was involved in the capsaicin-induced increase in cough sensitivity in guinea pigs. Furthermore, sinomenine partly reversed the effect of capsaicin. It was found that sinomenine significantly decreased the CRS and Penh values of guinea pigs. Besides, the production of SP and NKA were reduced after sinomenine treatment, which indicated that sinomenine diminished airway neurogenic inflammation and capsaicin-induced increase in cough sensitivity in guinea pigs.

Sinomenine, a bioactive compound extracted from *Sinomenium acutum*, a Chinese traditional medicinal plant, is used to prevent morphine dependence (Zhu et al., 2017; Lu et al., 2018). A previous study indicated that it attenuated inflammatory pain by regulating mTOR signals in anterior cingulate cortex (Li et al., 2017). Furthermore, sinomenine treatment blocked cell cycle and resulted in apoptosis in glioma cells (He et al., 2018). Sinomenine was reported to inhibit inflammatory response by regulating multiple targets. Sinomenine inhibited the expressions of iNOS, TNF- alpha, and COX-2, and reduced LPS-induced generation of superoxide anion and ROS in microglia (Qian et al., 2007). In addition, sinomenine mitigated osteoarthritis *via* inhibition of inflammation by acting on Nrf2/HO-1 signaling pathway (Wu et al., 2019). It was also reported that sinomenine suppressed the expressions of IL-1 and IL-6 in macrophages (Li et al., 2006; Zhou et al., 2008). In the present study, it was found that sinomenine attenuated airway neurogenic inflammation, at least in part, *via* alleviation of TRPV1 expression.

Activation of the ion channel of transient receptor potential (TRPV1) causes calcium ion influx into nerve terminals. Current research findings indicate that TRPV1 receptor is the “switch” for cough. The irritants that enter the lungs with air induce activation of sensory nerves on encountering the receptor, resulting in a series of reactions that manifest as cough. This study found that sinomenine reduced the protein level of TRPV1 in neuronal cells and decreased intracellular calcium concentration, leading to increased expressions and secretions of SP and NKA. A study reported that TNF-alpha sensitizes TRPV1 by promoting its expression (Wang et al., 2018). Moreover, it has been reported that ROS activated TRPV1 and enhanced glutamate release in neuron cells (Nishio et al., 2013). Furthermore, previous studies demonstrated that TRPV1 mediated the release of IL-1 and IL-6 (Sappington and Calkins, 2008; Obi et al., 2017; Yang et al., 2019). However, it is not known whether sinomenine inhibited the expression of TRPV1 *via* suppression of the signaling pathways related to production of TNF-alpha and ROS. This will be the focus of subsequent investigations.

Many organs and cell lines express SOX5, a transcription factor that belongs to the SoxD group of Sox family. It plays important role during developmental processes, and it influences cancer cell proliferation and metastasis (Hu et al., 2018; Sun et al., 2019). A previous study showed that SOX5 is also expressed in neurons (Lai et al., 2008). In addition, SOX5 mediated IL-6-induced RANKL upregulation in arthritic synovium (Feng et al., 2016). The results obtained in this study indicate that SOX5 transcription upregulated the expression of TRPV1 by capsaicin in neuronal cells. However, sinomenine pretreatment significantly suppressed the effect of SOX5 by inhibiting its expression. The mechanism through which sinomenine regulates the expression of SOX5 expression is still unknown. It is likely that sinomenine functioned as a ligand to the cell surface target receptor and activated downstream signal transduction, thereby regulating the expression of SOX5. This will be investigated in further studies.

## Conclusion

This study has demonstrated that atomized capsaicin stimulated high cough sensitivity in guinea pigs. However, sinomenine pretreatment attenuated the capsaicin-induced high cough sensitivity and reduced the infiltration of inflammatory cells. Furthermore, SOX5 promoted the expressions and secretions of SP and NKA, as well as the infiltration of monocytes/macrophages by transcriptional activation of the expression of TRPV1 gene. Sinomenine inhibited the expression of TRPV1, reduced intracellular calcium levels and secretions of SP and NKA, and reduced inflammatory cell infiltration *via* downregulation of protein expression of SOX5. These findings provide new insights into the treatment of aggravated cough sensitivity.

## Data Availability Statement

The original contributions presented in the study are included in the article/supplementary material, further inquiries can be directed to the corresponding author/s.

## Ethics Statement

The animal study was reviewed and approved by the Beijing University of Chinese Medicine.

## Author Contributions

J-LM, L-QS, N-NL, L-YW, S-JD, Y-XZ, and KJ conducted the experiments. S-HW, X-ML, YW, and J-YL designed the experiments and wrote the manuscript. All authors read and approved the manuscript.

## Conflict of Interest

The authors declare that the research was conducted in the absence of any commercial or financial relationships that could be construed as a potential conflict of interest.

## Publisher’s Note

All claims expressed in this article are solely those of the authors and do not necessarily represent those of their affiliated organizations, or those of the publisher, the editors and the reviewers. Any product that may be evaluated in this article, or claim that may be made by its manufacturer, is not guaranteed or endorsed by the publisher.

## Footnotes

1
https://www.bio-rad-antibodies.com/western-blot-protocol.html

2
https://www.genecards.org/
